# The Use of Red Mud in Agricultural Soil Cadmium Remediation: A Critical Review

**DOI:** 10.3390/toxics14010016

**Published:** 2025-12-23

**Authors:** Weiwei Sun, Wenyi Xie, Lei Wang, Lei Wang, Yang Gong, Xuwei Li, Chi Wang, Jiali Yan, Xiaochen Lin

**Affiliations:** 1 School of Civil Engineering and Architecture, Chuzhou University, Chuzhou 239000, China; sunweiwei9603@chzu.edu.cn (W.S.); aricher112@126.com (C.W.); 2 Nanjing Institute of Environmental Science, Ministry of Ecology and Environment of the People’s Republic of China, Nanjing 210042, China; wenyixie@foxmail.com (W.X.); leisureking@126.com (L.W.); gongyang@nies.org (Y.G.); lixuwei@nies.org (X.L.); 3 Ecological Environment Bureau of Chuzhou City, Chuzhou 239000, China; abc0878910544@163.com

**Keywords:** red mud, agricultural soil, Cd immobilization, soil remediation

## Abstract

Red mud is a highly alkaline solid waste with an annual emission of over 200 million tons, which requires large-scale utilization methods. Soil Cd remediation is a global concern, due to its high toxicity and strong mobility. Given red mud’s potential for soil Cd remediation, this study reviews its basic characteristics, the mechanisms of soil Cd immobilization by red mud, and the use of red mud-based passivators for agricultural soil Cd remediation. In general, red mud regulates soil pH, thus increasing the soil’s Cd adsorption capacity; provides abundant surface active sites for adsorption and complexation with soil Cd; introduces cations to immobilize Cd via ion exchange; and enriches Cd-resistant microbe species to reduce soil Cd toxicity. Furthermore, the potential environmental risks and suggestions on red mud application are discussed. Further research should focus on improving the remediation effectiveness of red mud on cadmium-contaminated agricultural soil, demonstrating its long-term efficacy and economic costs, and proposing practical technical models and standards for application.

## 1. Introduction

Red mud is a highly alkaline industrial by-product generated from alumina production. The yield of red mud varies depending on the alumina ore type and extraction process. In general, production of one ton of alumina yields 1 to 1.5 tons of red mud [1]. The annual global emissions of red mud exceed 200 million tons, and the estimated global reserves in 2024 are approximately 4 billion tons [2,3]. Most existing red mud is stored as slurry in piles, which requires a large amount of land. On the other hand, due to the high alkalinity and heavy metal content (such as chromium, copper, arsenic, cadmium, lead, molybdenum, and vanadium), the storage of red mud poses environmental problems, including water pollution, soil contamination, and air pollution. Moreover, red mud contains rare earth elements, including gallium, niobium, rhenium, scandium, tantalum, uranium, yttrium, and lanthanides; the insufficient recovery of valuable metals results in the underutilization of these resources [4,5,6]. The resource utilization and large-scale absorption of red mud have become a common focus of attention for both academic and industrial communities worldwide. Moreover, it is also a significant challenge that the global aluminum industry urgently needs to overcome for sustainable development.

At present, the principal approaches for the utilization of red mud primarily focus on the following aspects: (1) extraction of recovery of valuable metals, such as iron, aluminum, and rare metals [7,8]; (2) preparation of building materials, such as road base surface materials, cement, concrete, bricks, and ceramic granules [9,10,11]; (3) soilization of red mud, the preparation of artificial soil for vegetation restoration through the utilization of red mud [12,13,14,15]; (4) environmental contamination remediation, applying red mud as soil amendments, preparing desulfurization agents and wastewater adsorbents [16,17,18,19]. Among these utilization approaches, red mud has received increasing attention for the remediation of agricultural soil contamination with heavy metals [20].

With rapid industrial development, soil heavy metal pollution has become increasingly severe, posing a significant threat to ecosystems and human health [21]. Among various heavy metals, cadmium (Cd) is particularly of concern due to its high toxicity, strong mobility, and persistence [20,22]. It is reported that over 235 million hectares of agricultural soils worldwide are Cd-contaminated, especially in Asia and Europe [23]. The migration and accumulation of Cd in soil affect soil and crop quality and can enter the human body through the food chain, exerting detrimental effects on human nervous, reproductive, and respiratory functions [21,24]. Therefore, how to effectively remediate Cd-contaminated agricultural soil and reduce the environmental risks posed by Cd has become an important research topic.

The characteristics of red mud, including high alkalinity (pH 10–12.5), large specific area (15–30 m^2^·g^−1^), and rich iron (8–30.2%) and aluminum oxides (7.7–26.4%) contents, endow it with excellent Cd adsorption capacity, which therefore makes it a potential amendment for Cd-contaminated soil [20,25,26]. The utilization of red mud in Cd-contaminated agricultural soil has been widely reported; however, the influence of red mud on soil Cd mobility and plant Cd accumulation varies with experimental conditions [27,28]. This study reviews the mechanism of Cd-contaminated agricultural soil remediation by red mud and discusses the environmental risks of red mud application, aiming to provide references for agricultural soil Cd pollution remediation and the utilization of red mud.

## 2. Red Mud Characteristics

### 2.1. Red Mud Composition

Red mud is a solid waste generated during the alumina production process. Its physical and chemical properties, as well as its composition, vary depending on the bauxite and production technology. According to the alumina production process, red mud can be classified into sintering red mud, Bayer red mud, and combined red mud. The Bayer process for producing alumina, with its low energy consumption and high efficiency, has been widely adopted [14]. Table 1 summarizes the main composition of red mud generated by different production processes. In general, the main components of red mud are A1_2_O_3_, Fe_2_O_3_, SiO_2_, TiO_2_, CaO, and Na_2_O, accounting for 90%. The chemical compositions of different types of red mud vary significantly. The CaO content in red mud produced by the sintering and combined methods is higher, which is closely related to the addition of alkaline substances such as lime. On the other hand, Bayer red mud shows higher contents of aluminum and iron oxide. Moreover, red mud contains trace amounts of elements including chromium, copper, arsenic, cadmium, lead, molybdenum, and vanadium, as well as radioactive elements such as radium, thorium, and uranium [3,14].

### 2.2. Red Mud Physical and Chemical Properties

Red mud has a complex mineral composition, with loose microstructure and small particle size. There are significant differences in particle size distribution, pore structure, and chemical properties among red mud produced by different methods (Table 2). For example, Bayer red mud has a finer particle size (3–10 μm), large specific surface area, high alkalinity (pH 11.3 ± 1.0), poor permeability (permeability coefficient 10^−5^–10^−6^ m^2^·s^−1^), low shear strength (40–50 kPa), and high salt content (exchangeable sodium ions account for 53–91%) [34,35,36,37]. Compared with Bayer red mud, the sintering red mud exhibits a pH of 10–12, and possesses a larger particle size, ranging from 1 to 20 μm, with a maximum particle size of approximately 30 μm, which leads to a better permeability (permeability coefficient 10^−4^–11^−5^ m^2^·s^−1^) [20,29,35]. The sintering red mud also has a fine pore structure due to particle agglomeration of calcium carbonate and silicon dioxide crystallization [34].

## 3. Mechanisms of Soil Cd Remediation by Red Mud

### 3.1. Soil pH Regulation by Red Mud

Soil pH is a key factor that controls soil Cd environmental behaviors [38,39]. A decrease of one pH unit can result in a 4.3-fold increase in the solubility of Cd in soil [40]. On the contrary, a one-unit increase in pH (within 4–7.7) can lead to a 3-fold increase in soil Cd adsorption capacity [41]. As different studies involve varying soil types, the effect of pH on Cd solubility also differs. However, both studies suggest that higher pH values are favorable for Cd immobilization. Due to its high alkalinity, the application of red mud to soil significantly increases the soil pH, especially in acidic soils. And the increase in soil pH promotes the transformation of Cd^2+^ into insoluble precipitates, such as Cd hydroxides, carbonates, or phosphates [38,39,42], thereby decreasing soil Cd availability. Wang et al. found that 5% red mud increased soil pH by 0.5, with a maximum Cd stabilization efficiency of 68% [38]. In a severe Cd-contaminated soil, Gray et al. raised the soil pH by 1.68 with 5% red mud, and reduced the exchangeable Cd from 33 mg·kg^−1^ to 16 mg·kg^−1^ [42].

### 3.2. Adsorption and Complexation of Soil Cd by Red Mud

The aluminum and iron oxides in red mud provide abundant surface-active sites that can immobilize Cd ions through chemical adsorption and/or complexation. Li et al. found that the particle size of red mud-based passivator increased from 1–20 μm to 10–100 μm with an increase in Cd content from 0.21% to 3.48%, after the adsorption of Cd^2+^ [43]. The adsorption of Cd^2+^ by red mud involves both non-specific and specific adsorption [39,44,45]. Guo et al. demonstrated that both the Bayer red mud and sintering red mud followed the pseudo-second-order adsorption kinetics for Cd^2+^, and the adsorption maximum was 25.19 and 21.96 mg·g^−1^, respectively [46]. Furthermore, a study using extended X-ray absorption fine structure showed that Cd adsorption by red mud is mainly outer-sphere complexation (about 65%) and is accompanied by a smaller amount of inner-sphere complexation (about 35%) [47]. Luo et al. investigated the adsorption mechanism of red mud on Cd through X-ray near-edge adsorption spectroscopy, and indicated that Cd^2+^ and Cd(OH)Cl were the main components, and summarized the surface complexation model of Cd^2+^ sorbed to hydroxyl groups at neutral pH as reactions (1) and (2).

Cd^2+^ + SOH + H_2_O ↔ SOCdOH + 2H^+^ (1)

and/or

Cd^2+^ + SOH ↔ SOCd^+^ + H^+^ (2)

The outer-sphere complexation model is as reaction (3):

Cd^2+^ + 2SH ↔ CdS^2+^2H^+^ (3)

(SOH denotes the functional groups from iron and aluminum oxyhydroxide minerals, and SH represents ion exchange sites) [43,46,47].

### 3.3. Ion Exchange

The cations introduced by red mud (such as Na^+^ and Ca^2+^) may immobilize Cd via ion exchange [48]. Cd^2+^ in soil pore water can replace Ca^2+^ in red mud, thereby becoming immobilized. Meanwhile, the free Ca^2+^ could reduce the absorption of Cd by plants through ion competition [49]. Liu et al. demonstrated that applying red mud significantly increased the exchangeable calcium content in soil (by 31–78%) and decreased the available Cd [50]. However, an excessive amount of free cations may lead to Cd desorption. Zheng et al. found that overloading with CaCl_2_ and MgCl_2_ increased Cd release in the experimental soil by 11.2% and 10.8%, respectively [51]. Moreover, Yan et al. found that a low-dose red mud treatment (0.5%) increased the Cd release after the soil was flooded and then drained, due to the replacement of Cd sorbed on soil by Na^+^ at relatively low soil pH [52].

### 3.4. Microbial Mechanism

**Figure 1:**
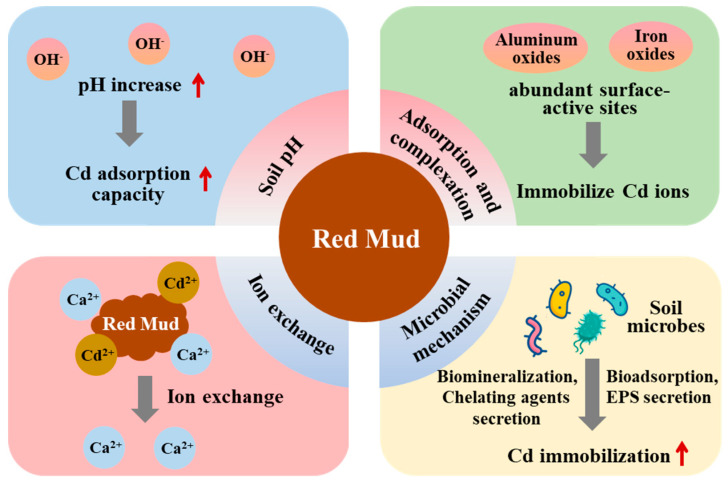
Immobilization mechanisms of Cd using red mud.

Soil microbes can reduce Cd toxicity and promote its immobilization through bioadsorption, biomineralization, and the secretion of extracellular polymers and chelating agents (Figure 1) [53,54]. In an acid paddy soil, the addition of red mud enriched specific soil bacterial phyla involved in iron reduction, which may contribute to the reduction in Cd release after soil drainage as well as soil Cd bioavailability [50]. In a vegetable garden soil, red mud promoted the proliferation of soil microbes and the formation of microbial biofilms, and immobilized Cd in the rhizosphere soil via biosorption and chemical chelation [55].

## 4. The Utilization of Red Mud-Based Passivator in Agricultural Soil for Cd Remediation

The immobilization of soil Cd by red mud is variable and highly dependent on soil and red mud properties. To improve its immobilization efficiency and control potential environmental risks, combined application and pre-treatment have been used to remediate Cd in agricultural soil using red mud.

### 4.1. Original Red Mud

The Cd immobilization effects by red mud varied with different application rates, and 0–6% red mud application rates are commonly used in existing studies [27,50,56,57]. The effects of original red mud on soil Cd availability and plant Cd accumulations with various application dosages are summarized in Table 3. At a relatively low application dosage, Yan et al. found that 2% red mud increased the pH of an acid paddy soil from 4.8 to 8.0, and reduced soil exchangeable Cd by 28% and rice grain Cd by 72% [50]. At higher application rates, Xu et al. found that 6% red mud increased soil pH from 4.7 to 7.5 and reduced exchangeable Cd by 24% [27]. Interestingly, red mud may have a reverse effect on Cd under certain conditions. Yan et al. flooded an acid paddy soil amended with 0.5% red mud, and found that during the beginning of the drainage, instead of lowering the Cd release, 0.5% red mud treatment increased the release of Cd from soil. While this phenomenon was not observed with 1% or 2% red mud treatments [52]. They assumed that the activation of Cd in the 0.5% red mud treatment group was highly related to the reductive dissolution of Fe(III) oxides. However, with higher red mud application rates, which resulted in higher soil pH, the influence of soil became the key factor and led to a decrease in Cd release [52].

### 4.2. Combined with Other Amendments

To further promote the soil Cd immobilization, many researchers studied the combined effects of red mud with other amendments (Table 4). Red mud and adsorptive materials such as zeolite can synergistically reduce the mobility of Cd in soil. Application of 5% composite material (red mud: zeolite = 3:1) decreased exchangeable Cd by 79.31% and carbonate-bound Cd by 45.17%, while increasing the residual Cd fraction by 170.84%. Moreover, the residual Cd and iron–manganese oxide-bound Cd fractions exhibited a significant negative correlation. The enhanced Cd immobilization may not only result from the adsorption effect of zeolite but also be associated with the substantial increase in available silicon in the soil induced by zeolite [59]. However, in another case, the combination of red mud with zeolite or gypsum did not reduce the soil Cd availability, perhaps due to the antagonistic effect of Ca^2+^ released from them with Cd^2+^ [48].

Red mud can also act synergistically with fertilizers to remediate Cd-contaminated agricultural soils. After 120 days of combined treatment with red mud and rice straw compost, the bioavailable Cd in the soil decreased by 75.3%, which was greater than the reduction achieved by red mud alone (68.4%) or compost alone (63.2%) [60]. Zhao et al. tested the effectiveness and longevity of red mud combined with rape or corn straw, and the results showed that with the combination of rape or corn straw, the reduction in soil available Cd increased from 35% to 42–48% in the first year, and 18% to 23–26% after eight years [61]. Besides organic amendments, combining red mud with inorganic materials also promotes soil Cd immobilization [62,63]. By combining red mud with inorganic fertilizer (silicon fertilizer and phosphorus fertilizer), Yang et al. reduced the soil available Cd by 47% and rice grain Cd by 62% [62]. Similarly, Li et al. demonstrated that applying red mud combined with lime and kaolin at various ratios reduced the soil available Cd by 28–57%, and the rice grain Cd by 47–82%, with 3 treatments lowering the rice grain Cd to below the food safety standard of Cd in China (0.2 mg kg^−1^) [63]. Besides inorganic amendments, combining red mud with organic materials also promotes soil Cd immobilization. For example, Zhao et al. increased soil pH by 25.5% in a field experiment using a combination application of red mud, biochar, and sepiolite [64]. As a result, the soil organic matter content increased by 34.6%, while the available Cd content decreased by 46.8%, leading to decreases in the transport coefficient of potato roots to tubers, roots to stems, and stems to leaves by 91.7%, 33.8%, and 33.3%, respectively [64]. These results predict greater soil Cd immobilization with combined application.

Many soil microbial strains have been reported to alter the availability of soil Cd [65,66,67]. Besides other amendments, the combination of red mud and specific microbes is expected to further enhance the Cd immobilization efficiency; however, relevant studies are still lacking. One example is that Zhu et al. applied red mud-Lactobacillus plantarum mixture to a Cd-spiked soil and lowered the soil available Cd and garlic bolt Cd contents with maximum rates being 60% and 44%, respectively [68].

**Table 4:** The combined effects of red mud with other amendments.

Amendments	Application Dosage	Soil pH Before Application	Soil Cd Concentration (mg·kg^−1^)	Available Cd Content After Application (mg kg^−1^)	Change in Extractable Cd Concentration (%)	Plant Cd Uptake (%)	Reference
Red mud, Silicon fertilizer, Phosphorus fertilizer	1800 kg ha^−2^ + 600 kg ha^−2^ + 675 kg ha^−2^	5.6	0.47	0.23	−47%	−63%	[62]
Red mud, Corn straw	0.5% + 0.1% (*w*/*w*)	8.9	1.13	0.58	−49%	-	[61]
Red mud, Rape straw	0.5% + 0.1% (*w*/*w*)	8.9	1.13	0.65	−42%	-	[61]
Red mud, Diatomite, Lime (5:3:2)	0.6 kg m^−2^	5.8	-	-	−29%	−59%	[69]
Red mud, Diatomite, Lime (5:3:2)	0.6 kg m^−2^	5.8	-	-	−72%	−72%	[69]
*Red mud*, *Astragalus sinicus* L. *straw*	22,500 kg hm^−2^ + 3000 kg hm^−2^	5.4	-	-	−25%	−45%	[70]
Red mud, *Astragalus sinicus* L. straw	22,500 kg hm^−2^ + 4500 kg hm^−2^	5.4	-	-	−31%	−55%	[70]
Red mud, Lime, Kaolin (7:1:4)	0.16%	5.5	1.28	0.76	−28%	−47%	[63]
Red mud, Lime, Kaolin (5:3:4)	0.16%	5.5	1.28	0.62	−41%	−49%	[63]
Red mud, Lime, Kaolin (4:4:4)	0.16%	5.5	1.28	0.59	−43%	−56%	[63]
Red mud, Lime, Kaolin (2: 6:4)	0.16%	5.5	1.28	0.45	−57%	−82%	[63]
Red mud, Biochar, Sepiolite (3:5:3)	5000 kg·hm^−2^	5.4	0.82	0.25	−47%	−87%	[64]

### 4.3. Red Mud Pre-Treatments

Pre-treatments before application are often used to either enhance red mud’s Cd sorption capacity and/or reduce environmental risks [25,71]. Multiple pre-treatment methods have been reported, including heat treatment, neutralization treatment, and ball milling treatment (Table 5) [25,71]. Heat treatment is conducted by calcinating dried red mud at 600–1000 °C, which leads to changes in its structure and phases and thereby improves red mud’s adsorption capacity [25]. Yang et al. demonstrated that 500 °C heat treatment significantly increased red mud’s specific surface area and nearly doubled Cd(II) sorption stability [71]. Neutralization treatments are usually conducted by mixing raw red mud with acid solutions (HCl, HNO_3_) or CO_2_, which greatly reduces the amount of alkali discharged into the environment [25]. Shi et al. found that reaction with 0.5% HCl increased the aluminum, calcium, and iron content on red mud’s surface and increased the number of adsorption sites [48]. As a result, this acid-treated red mud reduced soil available Cd by 28% [48]. Similarly, Luo et al. observed that after activation with hydrochloric acid (0.25 mol/L), the Cd^2+^ adsorption capacity of red mud increased from 0.16 mol·kg^−1^ to 0.19 mol·kg^−1^ (pH = 6.5). When combined with ball milling, the specific surface area rose markedly from 21.6 m^2^·g^−1^ of the raw red mud to 77.4 m^2^·g^−1^, exposing more adsorption sites and further enhancing the Cd adsorption capacity to 0.21 mol/kg. Moreover, the adsorbed Cd exhibited greater stability, with a decrease in the proportion of exchangeable Cd and an increase in the residual Cd fraction [39]. Compositing nanoparticles onto red mud can also enhance its Cd adsorption capacity. Pang et al. employed a redox precipitation method to deposit nanoscale MnO_2_ onto red mud, increasing its specific surface area from 10.22 m^2^·g^−1^ to 38.91 m^2^·g^−1^ and its pore volume from 0.02 cm^3^·g^−1^ to 0.73 cm^3^·g^−1^, thereby nearly tripling its Cd adsorption capacity to 46.36 mg·g^−1^ [72]. However, most studies on treated red mud are conducted in liquid systems to investigate their Cd adsorption capacity [25,71]. The efficiency and environmental risks of treated red mud for Cd remediation in agricultural soil, particularly its effects on plant Cd accumulation, warrant further study.

## 5. Environmental Risks of Red Mud Application

Red mud utilization is a top research topic and has made significant progress in the remediation of Cd-contaminated soils. However, the toxic heavy metals, radioactive elements, and strong alkalinity of red mud pose potential environmental risks when applied to agricultural soils.

### 5.1. Toxic Heavy Metals in Red Mud

Red mud contains toxic heavy metals, such as chromium, copper, arsenic, cadmium, lead, molybdenum, and vanadium. The improper application of red mud may lead to the release of these elements into the soil and groundwater systems through rain leaching, causing secondary environmental pollution [73,74]. A soil column experiment simulating the situation after the Hungarian red mud disaster showed that the leaching solution of the red mud significantly changed the chemical properties of the soil, and the concentrations of various soluble elements, such as Cr, Cu, Na, Mo, and Pb in the soil increased after red mud treatment [75]. Studies by Ujaczki et al. have shown that excessive application of red mud poses environmental risks and reduces crop yields. When red mud application exceeded 20%, the total As, Cr, and Ni in the soil reached 21, 98, and 46 mg kg^−1^, respectively [76]. Furthermore, a 30% red mud application resulted in a 50% decrease in white mustard growth [76]. As for Cd, most of the red mud contains a relatively low Cd. Hua et al. summarized the Cd content in red mud from different countries, and all red mud Cd content ranges from below LOD to 34 mg kg^−1^ dry weight, which is within the EU maximum permissible limits for metals/metalloids in sludge for application to agricultural land (20–40 mg kg^−1^), except for one case in Brazil (86 mg kg^−1^) [77]. When using red mud, attention should be paid to its inherent heavy metal contents and their leachability when mixed with soil. It is necessary to limit the application rate to prevent potential soil contamination.

### 5.2. High Alkalinity of Red Mud

Red mud contains a large amount of alkaline substances, with a relatively high pH value of 10–12, and the application of red mud in soils usually increases its pH [78,79,80]. Besides the effects on Cd, significant changes in soil pH also affect nutrient availability, thereby influencing plant nutrient absorption and growth [81,82]. Liang et al. demonstrated that when 1% red mud was applied to soil, the release of phosphorus decreased from 14.4 to 2.6 mg·kg^−1^ [83]. Kong et al. reported that red mud promoted the growth of salt marsh species, *Spartina alterniflora*, but not that of freshwater marsh species, *Sagittaria lancifolia* [84]. Attention should be paid to the alignment of soil pH changes with the requirements of the target crops.

### 5.3. High Sodium Content in Red Mud

The Na_2_O content in red mud can reach 10%, and long-term or excessive application of red mud can significantly increase soil electrical conductivity, leading to soil salinization [85,86]. When red mud application was 40–50%, the sodium content reached 100 times that of the control group [76]. An increase in the exchangeable sodium content in the soil will disrupt the structure of soil aggregates, resulting in soil compaction, diminished water and air permeability, and, subsequently, affecting plant roots’ water and nutrient absorption and plant growth [86]. Measures should be taken to prevent soil salinization. If necessary, red mud should undergo dealkalization pretreatment to remove sodium and other alkali substances.

### 5.4. Effect of Red Mud on Soil Microbes

Soil microbes are involved in ecosystem processes such as nutrient cycling and the decomposition of organic matter, and play a crucial role in maintaining the health and ecological functions of soil [87]. Changes in soil pH following red mud application may alter the diversity and composition of soil microbial communities [87,88]. Although Yan et al.’s study showed that 2% red mud application had little impact on soil microbial alpha diversity and even improved the stability of the co-occurrence network [50], higher rates may have adverse effects. Feigl et al.’s study showed that 30–50% red mud application lowered the soil bacterial Shannon index by 18–48% [89]. On the other hand, it is reported that 4% red mud application significantly reduced the abundance of heterotrophic fungi in the soil and caused the bacterial community to shift from being dominated by Gram-positive bacteria to being dominated by Gram-negative bacteria, and even led to the disappearance of specific key microbial populations (such as the Arthrobacter genus). Meanwhile, red mud reduced the activity of soil enzymes, including β-glucosidase, which plays a vital role in the soil carbon–nitrogen cycling [90]. The decline in the activity of these enzymes indicated the degradation of soil ecological functions. Currently, the interaction mechanisms between red mud and microorganisms remain insufficiently understood and require further investigation.

## 6. Outlook on Remediation of Cadmium-Contaminated Agricultural Soil Using Red Mud

Although red mud shows excellent potential for agricultural soil Cd remediation, most existing studies have been conducted in simulation experiments under controlled conditions, which may differ significantly from large-scale application in a real environment. In addition to the environmental risks discussed above, other concerns arise when red mud is applied to agricultural soils.

(1) The specificity and uncertainty of the remediation effect

The effect of Cd remediation by red mud is significantly affected by many factors, such as red mud composition, soil properties, Cd concentrations, co-existing metals, and plant types [77,85]. For instance, red mud showed a better Cd immobilization effect than zeolite in calcareous soil, but its performance differed in acidic soil [77]. Furthermore, the application dosage is also a crucial factor. A proper amount of red mud may benefit plant growth, whereas excessive application may, on the contrary, inhibit it due to salinization or ionic toxicity. Due to the diversity of red mud and soil properties, there is no “one-size-fits-all” standardized application strategy. Before each remediation, a detailed site-specific risk assessment must be conducted, including the physical and chemical properties of the soil and the red mud used, to determine the optimal pre-treatment method and application dosage [85]. This not only increases initial research costs and time, but also raises the requirements for the technicians.

(2) The long-term effects of red mud on soil Cd immobilization

Although red mud can effectively immobilize soil Cd through mechanisms such as increasing soil pH, precipitation, adsorption, and complexation, the long-term stability of these effects remains unclear [85,86]. Agricultural soil is a dynamic system with its physical and chemical properties changing over time. Under the influence of factors such as long-term rainfall leaching, organic acids secreted by plant roots, or the application of acidic fertilizers, the remediated soil by red mud may be re-acidified, leading to the reactivation and release of heavy metals and causing secondary environmental risks. Moreover, changes in soil redox potential also affect the Cd immobilization. For example, the periodic alternation of flooding and drainage conditions in paddy soil systems causes the dissolution and regeneration of iron oxides, which control the release of Cd, and in turn affect the immobilization of Cd by red mud [52]. However, currently, there are still very scarce field test data on the long-term stability of red mud in Cd immobilization [25].

(3) The economic cost of soil Cd remediation by red mud

The economic cost of red mud remediation is a key factor that limits its large-scale application. Although red mud, as an industrial waste, is almost cost-free, its application process involves multiple high-cost steps. Firstly, the transportation cost may be high. Red mud is a large, high-density bulk material, and its sources are concentrated in aluminum smelting plants, while contaminated agricultural soils may be located far away. Transporting hundreds of thousands of tons of red mud over long distances to the remediation site will incur huge transportation costs [85]. Secondly, the pre-treatment cost significantly increases the total cost. When applied to neutral or alkaline soil, to avoid the excessive import of alkalinity and enhance remediation efficiency, complex pretreatment processes, such as acid neutralization, physical modification, or compounding with other materials, are often required [68,91]. Although these treatments can enhance remediation, they also increase costs. Most current research lacks a comprehensive cost–benefit analysis of the red mud remediation. Compared with environmental and social benefits, the direct economic profit generated by using red mud to remediate agricultural soil may be relatively low. A complete economic feasibility assessment should comprehensively consider materials, transportation, pre-treatment, application, long-term monitoring, and potential environmental risk costs. However, data in this aspect is extremely scarce.

## 7. Conclusions

Red mud has exhibited significant potential as a cost-effective and efficient amendment for the remediation of Cd-contaminated agricultural soils. Its high alkalinity, large specific surface area, and abundant iron and aluminum oxides collectively contribute to cadmium immobilization through multiple mechanisms, including pH regulation, adsorption, complexation, ion exchange, and microbial interactions. Furthermore, the application of red mud can take various forms—it may be directly incorporated into soils, combined with other amendments or microbial agents, or utilized following specific pre-treatments. In addition, the use of modified or composite red mud materials has been shown to further enhance Cd stabilization and reduce environmental risks. However, the remediation performance of red mud remains highly dependent on its physicochemical properties, soil characteristics, application forms and surrounding environmental conditions. Concerns also persist regarding the long-term stability of immobilized Cd, potential secondary pollution, and the economic feasibility of large-scale applications. Therefore, future studies should focus on field-scale verification, quantitative assessment of long-term stability, comprehensive environmental risk evaluation, and cost–benefit analyses. Establishing standardized technical guidelines and safe application frameworks will be essential for promoting the sustainable and large-scale utilization of red mud in agricultural soil Cd remediation.

## Figures and Tables

**Table 1:** Red mud composition from different production processes.

Red Mud Type	Origin	Composition %								Reference
		SiO_2_	Fe_2_O_3_	Al_2_O_3_	CaO	MgO	TiO_2_	Na_2_O	K_2_O	
Bayer	Guizhou, China	8.5	26.4	19.0	21.8	-	7.4	4.8	0.07	[29]
	Brazil	12.2	34.5	22.1	3.8	-	3.6	5.3	0.7	[30]
	Turkey	17.3	35.0	20.2	5.3	0.3	4.0	9.4	-	[31]
	Russia	12.04	53.21	13.3	5.98	-	5.48	5.16	-	[32]
sintering	Guizhou, China	17.3	10.4	10.4	40.2	-	7.1	3.5	0.05	[29]
	Russia	12.05	40.10	12.8	8.40	-	4.10	7.82	-	[32]
	Russia	4.0	16.6	34.8	4.8	0.8	1.80	33.0	-	[33]
combined	Chongqing, China	24.4	8.5	21.7	25.5	1.2	3.7	6.9	2.8	[34]

**Table 2:** Properties of red mud from different production processes.

Red Mud Type	pH	Particle Size Range (μm)	Permeability (m^2^·s^−1^)	Shear Strength (kPa)	Exchangeable Sodium Content (%)	Reference
Bayer	11.3 ± 1.0	2–18	10^−5^–10^−6^	40–45	53–91	[20,29,37]
Sintering	10–12	1–20	10^−4^–11^−5^	287	-	[20,29,37]

**Table 3:** Effects of red mud on cadmium accumulation in plants.

Red Mud Addition Rate (% *w*/*w*)	Red Mud pH	Cd Concentration in Soil (mg·kg^−1^)	Soil pH After Red Mud Addition	Available Cadmium (Cd) Content After Red Mud Addition (mg kg^−1^)	Change in Extractable Cd Concentration (%)	Plant Uptake Cd Change (%)	Reference
0.5%	11.1	1.5	-	-	−46.7%	−36.7%	[58]
1%	11.7	50.5	4.80/6.80	37.3	−26.14%	-	[50]
1%	-	-	4.84/6.10		−10%	-	[27]
2%	11.7	50.5	4.80/8.00	36.4	−27.92%	−75%	[50]
4%	-	-	4.84/7.87	-	−19%	-	[27]
4%	12.5	1.79	7.13/11.29	1.33	−25.70%	-	[56]
5%	-	4.36	6.71/7.87	2.90	−33.34%	−48.01%	[57]
6%	-	-	4.84/8.13	-	−24%	−49.68%	[27]

**Table 5:** Red mud pre-treatments.

Pre-Treatment	Description	Effects
Heat treatment	Calcination at 600–1000 °C	Changes red mud’s structure and phases and significantly enhances red mud’s Cd adsorption capacity.
Neutralization treatment	Raw red mud is mixed with acid solutions (HCl, HNO_3_) or CO_2_	Reduces the amount of alkali discharged into the environment.
Ball milling treatment	Ball milling	Specific surface area exposes additional adsorption sites, resulting in a marked enhancement of Cd immobilization.

## Data Availability

Not applicable.
